# Long Term Survival in a Patient Following Resection for Carcinoma of the Gallbladder and Rectum

**DOI:** 10.1155/1993/84854

**Published:** 1993

**Authors:** S. B. Kelly, N. A. Habib, L. H. Blumgart

**Affiliations:** 1 Department of Surgery Royal Postgraduate Medical School Hammersmith Hospital London UK; 2 Klinik fur Viszerale und Transplantationschirurgie Universitat Bern Inselspital Bern CH-3010 Switzerland; 3 Department of Surgery Royal Postgraduate Medical School Hammersmith Hospital Du Cane Road London W12 0NN UK

## Abstract

We describe a 66-year-old man who presented initially with acute cholecystitis. He was treated by cholecystostomy and biopsy of the gallbladder mucosa which revealed carcinoma of the gallbladder. Four weeks later a cholecystectomy was performed followed by resection of the common bile duct, common hepatic duct and segments IV and V of the liver and a hepaticojejunostomy. Sixteen months later an abdomino-perineal resection was performed for a moderately differentiated Dukes′ stage C carcinoma of the rectum. He is alive and without evidence of recurrence seven years later. Few patients survive for this length of time following resection of either carcinoma of the gallbladder or rectum. This case report demonstrates the value of aggressive surgical treatment in patients with early carcinoma of the gallbladder.


*HPB Surgery*, 1993, Vol. 7, pp. 165–174
Reprints available directly from the publisher
Photocopying permitted by license only
© 1993 Harwood Academic Publishers GmbH
Printed in the United States of America

### CASE REPORT

### LONG TERM SURVIVAL IN A PATIENT FOLLOWING RESECTION FOR CARCINOMA OF THE GALLBLADDER AND RECTUM

S.B. KELLY, N.A. HABIB* and L.H. BLUMGART**
*Department of Surgery, Royal Postgraduate Medical School, Hammersmith Hospital, London, and Klinik fur Viszerale und Transplantationschirurgie**, Universitat Bern, CH-3010 Bern, Inselspital, Switzerland*

*(Received 19 August 1992)*

We describe a 66-year-old man who presented initially with acute cholecystitis. He was treated by cholecystostomy and biopsy of the gallbladder mucosa which revealed carcinoma of the gallbladder. Four weeks later a cholecystectomy was performed followed by resection of the common bile duct, common hepatic duct and segments IV and V of the liver and a hepaticojejunostomy. Sixteen months later an abdomino-perineal resection was performed for a moderately differentiated Dukes' stage C carcinoma of the rectum. He is alive and without evidence of recurrence seven years later. Few patients survive for this length of time following resection of either carcinoma of the gallbladder or rectum. This case report demonstrates the value of aggressive surgical treatment in patients with early carcinoma of the gallbladder.

KEY WORDS: Gallbladder neoplasms, rectal neoplasms

### INTRODUCTION

Carcinoma of the gallbladder is the commonest form of biliary tract malignancy and the fifth most common gastrointestinal cancer, pancreatic cancer occurring about five times as frequently. The disease is encountered in 1–2% of cholecystectomy specimens and caused the death of over 2,000 men and 3,500 women in England and Wales between 1966 and 1970 (Cancer Mortality England and Wales, 1911–1970)[1].

The disease is uniformly associated with a rapidly lethal course independent of any form of treatment. This reputation largely stems from the early silent growth of the tumour with the late presentation and the great difficulty in adequately excising tumours in the region of the porta hepatis. Piehler and Crichlow (1978)[2], in a collective literature review, found an incidence of gallbladder carcinoma in operations on the biliary tract of 1.91% (range 0.55%–6.50%). They reported an overall 1-year survival rate of 11.8% and a 5-year survival rate of 4.2%.

Address correspondence to: Mr N.A. Habib, Department of Surgery, Royal Postgraduate Medical School, Hammersmith Hospital, Du Cane Road, London W12 0NN, UK

### CASE REPORT

A 66 year old man initially presented with an 18 hr history of severe right sided abdominal pain. He had vomited once and he had lost one stone in weight. His past medical history included diverticular disease (confirmed by barium enema) and he had been passing blood rectally for several months. On admission, he had a temperature of 38 °C, a pulse rate of 80 beats/min and a blood pressure of 150/90 mm Hg. Abdominal examination revealed localised tenderness, guarding and rebound in the right hypochondrium. White cell count was $8.2 \times 10^9$/l.

Laparotomy revealed an acutely inflamed and grossly distended gallbladder with purulent fluid in the pelvis, marked diverticular disease of the sigmoid colon, but no evidence of perforation. After opening the gallbladder, ten large facetted stones and sludge were removed and the lining of the gallbladder was curetted and the tissue sent for histology. A cholecystostomy was then performed using a 16 Fr. malecot catheter. Histology revealed a moderately differentiated adenocarcinoma of the gallbladder. After recovering from the operation, he was transferred to the Hammersmith Hospital for further investigation and treatment.

CAT scan revealed a normal liver and questionable irregularity of the gallbladder wall (Figure 1). A tubogram performed via the cholecystostomy tube revealed sludge in the gallbladder and two large radiolucent stones, one in the mid common bile duct and one in the common hepatic duct (Figure 2). The common bile duct was dilated at 1.6 cm dia. The left intrahepatic ducts could not be filled, however, the right hepatic ducts were not dilated and there was free flow of contrast into the duodenum. A visceral angiogram was normal.

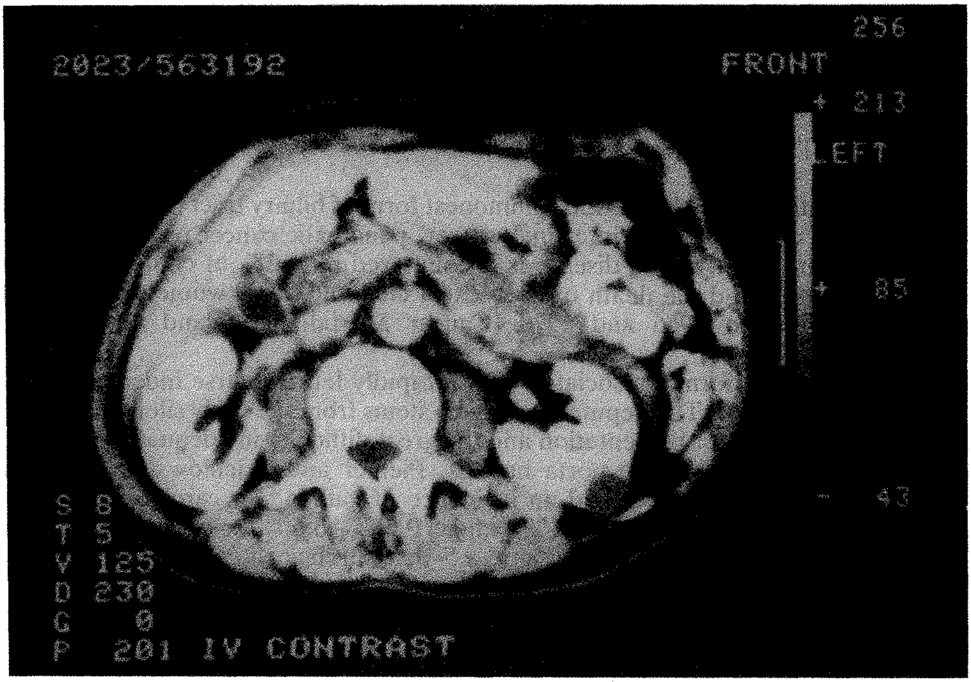

**Figure 1** CAT scan demonstrating questionable irregularity of the gallbladder wall and a normal liver.

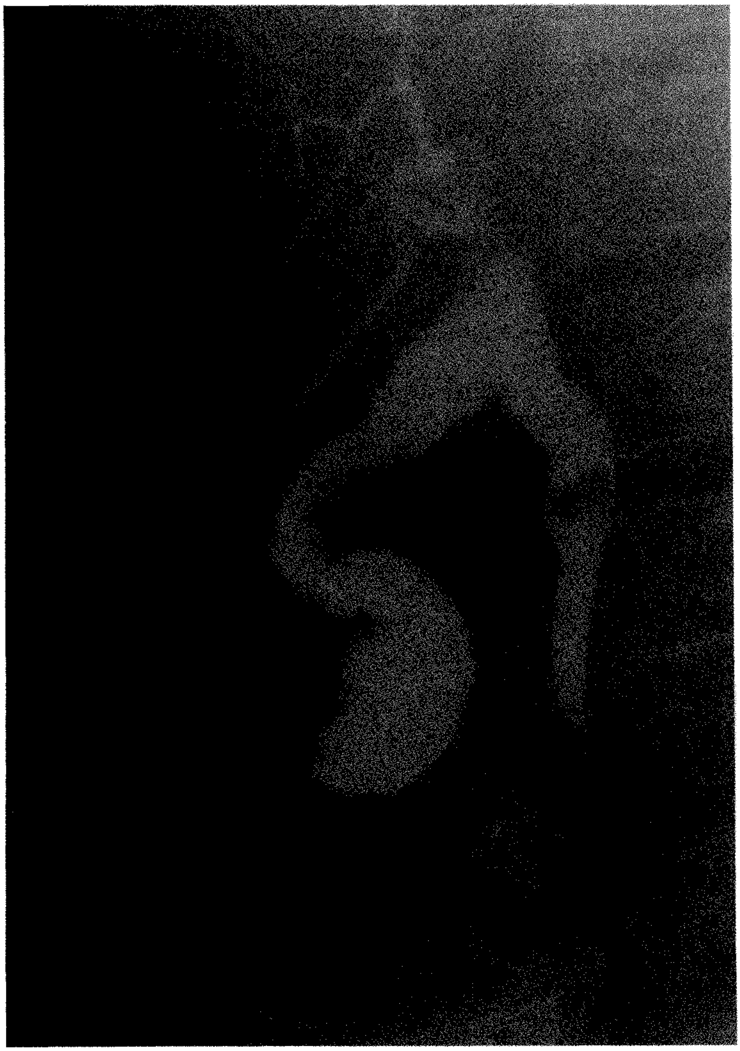

**Figure 2** Tubogram demonstrating sludge in the gallbladder and two large radiolucent stones, one in the common bile duct and the other in the common hepatic duct.

At laparotomy, a palpable mass was found in the fundus of the gallbladder and there was evidence of chronic cholecystitis. No stones were palpable in the gallbladder, however two large stones were palpable in the common bile duct. Two enlarged lymph nodes were present in the region of the cystic duct but these did not appear to be involved with tumour and there was no evidencve of gross invasion of the liver.

The cholecystostomy tube was removed and the tract through the abdominal wall excised. The common bile duct was transected above the duodenum and the distal end closed. Then the entire common bile duct and common hepatic duct were turned upwards anterior to the portal vein taking lymph nodes and all tissue anterior to the portal vein upwards and forwards. The right hepatic artery crossed anterior to the common hepatic duct and had to be sacrificed to obtain clearance. The common hepatic duct was transected at the confluence and segments IV and V of the liver and the gallbladder were removed. A clearance of 2–3 cm of liver tissue was achieved on all sides. Finally, a hepaticojejunostomy was performed. Histological examination confirmed a well differentiated adenocarcinoma of the gallbladder invading to the level of the muscularis but with no serosal or liver invasion. A mixed stone (0.8 cm dia.) was present at the orifice of the cystic duct. There was no involvement of the cholecystostomy tract by tumour. A tubogram performed one week postoperatively revealed normal calibre bile ducts. No filling defects were noted and there was good flow of contrast into the jejunum (Figure 3). Postoperative recovery was complicated by a pelvic abscess which drained spontaneously per rectum.

Sixteen months later he was investigated for left iliac fossa pain, tenesmus and the passage of blood and mucus per rectum. On rectal examination he was found to have an ulcer on the anterior wall of the rectum, 6 cm from the anal verge. Sigmoidoscopy revealed an irregular polypoid lesion in the mid rectum, biopsy of which demonstrated a moderately differentiated adenocarcinoma. Barium enema revealed a small area of irregularity in the rectal wall anteriorly at the recto-sigmoid junction and extensive diverticular disease of the sigmoid colon. An ultrasound scan of the liver was clear. Following this an abdomino-perineal excision of rectum was performed. Histology revealed a moderately differentiated carcinoma of the

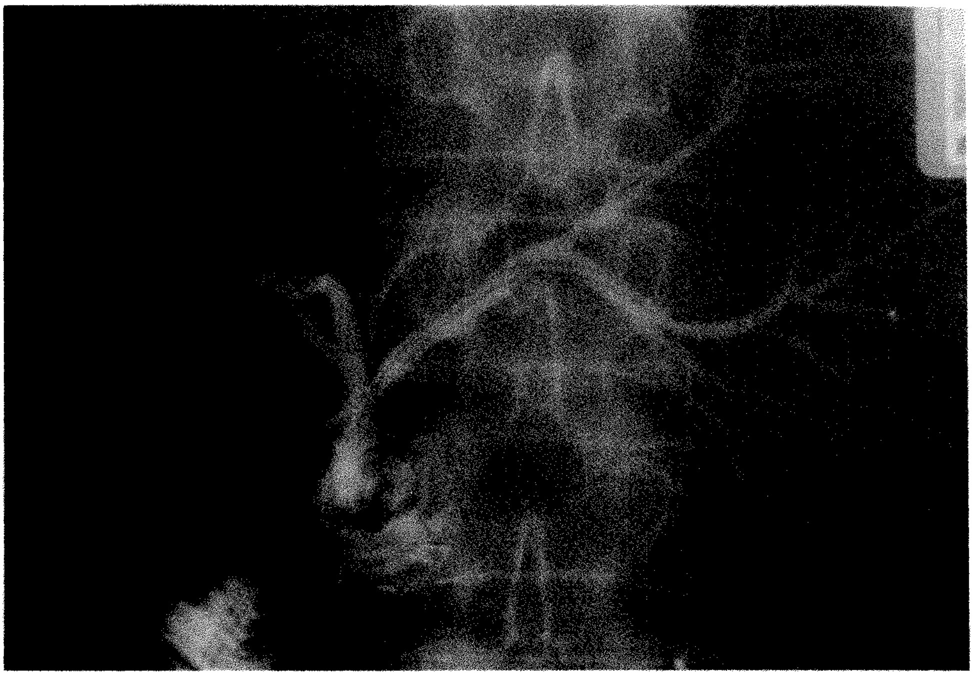

**Figure 3** Postoperative tubogram demonstrating normal calibre bile ducts with good flow of contrast from the bile ducts into the jejunum.

rectum, Dukes' stage C. In addition, three polyps were found, two of which were tubulovillous adenomas with moderate dysplasia and the third showed moderately differentiated adenocarcinoma extending into the submucosa (Nevin stage 2)[3].

At present, he is alive and well, without evidence of recurrence seven years following hepatectomy for gallbladder carcinoma and six years after abdomino-perineal excision for adenocarcinoma of rectum.

### DISCUSSION

Patients living more than five years following the diagnosis of gallbladder carcinoma are usually those in whom the cancer was an unexpected histological finding following cholecystectomy for presumed benign disease. Most series report 5-year survival rates for patients with primary carcinoma of the gallbladder of less than 5%. Furthermore, the 5-year survival rate for patients with Dukes' stage C colorectal cancer is 12–27%[4,5]. It is therefore surprising that our patient survived for so long. Controversy exists as to whether simple cholecystectomy is sufficient or whether these patients should undergo more radical surgery with hepatic resection and dissection of regional lymph nodes. Aldridge *et al.* (1990)[6] suggest that bisegmentectomy (IV/V) and node dissection offer the chance of cure in a selected group of patients and worthwhile palliation in others. They reported a group of 137 patients who underwent surgical treatment of gallbladder cancer. Curative resection (cholecystectomy plus bisegmentectomy (IV/V) and node dissection) was performed in 14 (10%). Eight of these patients had no residual disease following surgical treatment and all were alive and without recurrence at between 15 months and 11 years follow-up.

Morrow *et al.* (1983)[7] reported that of 11 of 112 patients (10%) with primary gallbladder cancer with tumour limited to the gallbladder wall (stages 1 to 2), one of five patients (20%) treated with cholecystectomy alone and four of six patients (67%) treated with cholecystectomy and lymphadenectomy (with hepatic wedge resection in three and pancreaticoduodenectomy in one) were alive and disease-free three to six years after operation. They concluded that although the patient numbers in each group were small, the survival rate in this subgroup was higher than the rate in patients with stages 1 to 2 disease treated with cholecystectomy alone and it seems appropriate to be aggressive in view of the natural history of the disease.

Gallstones have been found in 70–98% of cases of gallbladder cancer and are thought to predispose to carcinoma by chronic trauma and inflammation of the gallbladder mucosa leading to dysplastic changes and carcinoma. The relative risk of carcinoma is increased when the symptoms and signs of cholecystitis have previously occurred[8] as in this case. Histologically, cholecystitis is usually present in association with carcinoma and when chronic cholecystitis has led to gallbladder calcification, the risk of malignancy is much increased[9]. The risk of cancer in the porcelain gallbladder is very high and justifies prophylactic cholecystectomy[10]. Similarly, patients with retained gallbladders following cholecystectomy are at a demonstrably increased risk[11,12].

Carcinoma of the gallbladder spreads mainly by lymphatics and by direct extension into the liver substance. The most logical surgical approach for a carcinoma of the gallbladder would consist of an operation that would accomplish total removal of the organ and the gallbladder bed (an adequate wedge of underlying liver) and a node dissection of the draining lymphatics[13,14]. This so-called "extended cholecystectomy" involves en bloc removal of the adventitia and contained lymphatics surrounding the bile duct, portal vein and hepatic artery. The limits of this dissection extend from the nodes behind the first and second parts of the duodenum and the head of the pancreas, across to the coeliac axis and then extend upwards to the base of the liver and the porta hepatis. The recommended extent of the resection of liver substance has ranged from a non-anatomical wedge resection of the gallbladder bed to formal removal of segments IV and V including the gallbladder fossa[15,16] and even to right hepatectomy. Wedge excision, although appearing to be the least radical of these procedures, is often difficult and complicated by significant blood loss because of the non-anatomical dissection. Right hepatectomy is probably not warranted when the tumour is confined to the gallbladder bed and is unlikely to prolong survival when more extensive invasion is present[17]. A segmental liver resection can be carried out based on a precise knowledge of the anatomical organization of the liver and appears to be the best option if liver resection is indicated.

### *References*

1. Cancer mortality England and Wales 1911–1970. Studies on medical and population subjects, No. 29. London: HMSO
2. Piehler, J.M. and Crichlow, R.W. (1978) Primary carcinoma of the gallbladder. *Surgery Gynecol. & Obstet.*, **147**, 929–942
3. Nevin, J.E., Moran, T.J., Kay, S. and King, R. (1976) Carcinoma of the gallbladder. Staging, treatment and prognosis. *Cancer,* **37**, 141–148
4. Gill, P.G. and Morris, P.J. (1978) The survival of patients with colorectal cancer treated in a regional hospital. *Br. J. Surg.*, **65**, 17–20
5. Armitage, N.C. and Hardcastle, J.D. (1985) Screening for colorectal cancer. *Eur. J. Surg. Oncol.*, **11**, 327–331
6. Aldridge, M.C., Castaing, D. and Bismuth, H. (1990) Gallbladder cancer: Resection for cure? *HPB Surgery*, **2 (supp)**, 12
7. Morrow, C.E., Sutherland, D.E.R., Florack, G., Eisenberg, M.M. and Grage, T.B. (1983) Primary gallbladder carcinoma: significance of subserosal lesions and results of aggressive surgical treatment and adjuvant chemotherapy. *Surgery*, **94**, 709–714
8. Broden, G. and Bengtsson, L. (1980) Carcinoma of the gallbladder, its relation to cholelithiasis and to the concept of prophylactic cholecystectomy. *Acta. Chir. Scand. Supp.*, **500**, 15–18
9. Polk, H.C. (1966) Carcinoma and the calcified gallbladder. *Gastroenterology*, **50**, 582–585
10. Berk, R.N., Armbuster, T.G. and Saltzstein, S.L. (1973) Carcinoma of the porcelain gallbladder. *Radiology*, **106**, 29–31
11. Cowley, L.L. (1964) Carcinoma of the gallbladder following cholecystectomy. *Ann. Surg.*, **30**, 474–475
12. Cowley, L.L. and Wood, V. (1964) Carcinoma developing in a remnant of the gallbladder. *Ann. Surg.*, **159**, 465–468
13. Adson, M.A. (1973) Carcinoma of the gallbladder. *Surg. Clin. N. Am.*, **53**, 1203–1216
14. Fahim, R.B., McDonald, J.R., Richards, J.C. and Ferris, D.O. (1962) Carcinoma of the gallbladder: a study of its modes of spread. *Ann. Surg.*, **156**, 114–124
15. Bismuth, H., Houssin, D. and Castaing, D. (1982) Major and minor segmentectomies 'reglees' in liver surgery. *World J. Surg.*, **6**, 10–24
16. Blumgart, L.H. (1984) Tumours of the gallbladder and bile ducts. In: *Maingot's abdominal operations*, edited by H. Ellis, 8th edn, Schwartz S.I., Appleton-Century Crofts
17. Bismuth, H. and Malt, R. (1979) Carcinoma of the biliary tract. *New Engl. J. Med.*, **301**, 704–706

*(Accepted by S. Bengmark 30 September 1992)*

### INVITED COMMENTARY

I remember how I felt in 1961, when I read Richard Brasfield's report: "Right Hepatic Lobectomy for Carcinoma of the Gall Bladder: A Five Year Cure"[1]. Thirty years later the same feeling of being disconnected from a peculiar line of reasoning returned as I read Hammersmith's report above. As Yogi Bera said: "Its deja vu all over again."

Let me explain. In both instances, surgeons have attributed their patients' good fortune to unnecessarily large operations, rather than to the natural history of the patient's very small tumors; and in both reports the risk of extended operations has been ignored. Brasfield's report in no way justified removal of hepatic segments IV through VIII as an "opportunity for cure" of tumors at "an early stage" when direct hepatic invasion was of microscopic extent[2]. In the same way the case report above does not, as the authors claim, "demonstrate the value of aggressive surgical treatment in patients with very early carcinoma of the gall bladder" —specifically, not for this TlaNoMo tumor which would have been cured by cholecystectomy alone.

The effect of such unsupported claims upon mature or skeptical surgeons is negligible. However, obligations of writing[4] must be fulfilled when young surgeons, who may be more easily misled, look to the authoritative surgical authors of the world as guides to proper surgical aggression. One year after the 1961 account, Brasfield and Pack reported seven hepatic lobectomies done for gallbladder cancer; there was a single five-year survivor and four operative deaths[5].

The disparity between risk and possible benefit is less obvious in the case now at hand. Nevertheless, most readers would like to know whether it was necessary to remove the common bile duct "to obtain clearance" when there was no gross evidence of liver or ductal involvement, as a consequence, how division of the right hepatic artery might increase the risk of hepatojejunostomy done in combination with hepatic resection. These questions are relevant when in this circumstance wedge resection of the gallbladder bed, however non-anatomical, (combined with regional lymphadenectomy) involves little risk, will give margins free of tumor when liver invasion is not grossly evident, and may be done safely by most surgeons without need for referral or cost of a second operation.

The tumor described in this case report was too early to demonstrate the value of aggressive surgical treatment. Nevertheless, there are good reasons to be aggressive—to remove more than the gallbladder when resecting early carcinomas of that origin. The questions are: How early? And how aggressive? This concerns the relationship between the first step a cancer takes in growing beyond the gallbladder, and the first step a surgeon can take *safely* to remove more than the gallbladder. *Earliness* considered in this way includes: the 10 percent of cancers that are confined to the gallbladder (Tl-T2), and the 15 percent that either invade just a short way into the adjacent liver (T3), or involve only first order regional lymph nodes (N1). In this light, the first order of surgical *aggressiveness* concerns these sites of spread that can be removed safely by most abdominal surgeons.†

Until very recently there has been no way to report correlations of surgical aggressiveness with tumor stage, because no classification of tumor extent had

†There is more controversy about the risk and benefit of aggressive resection of more extensive (non-early) tumors that will not be considered here.

surgical relevance: lymphatic involvement was seen as "any nodes" — near or far — instead of lymph nodes that might be safely removed; and hepatic involvement was not considered in relation to its removability. But now, the TNM staging system referred to above (the 1992 American Joint Committee on Cancer Classification, has been modified from earlier editions to reflect stages that have true surgical relevance[3]. Specifically, T1 and T2 tumors are those which have not grown through the serosa or into the liver. T3 lesions are those that extend 2 cm. or less into the liver. N1 denotes metastases in cystic duct, pericholedochol and/or hilar lymph nodes.

(This stratification of first order lymph node involvement (N1) puts less surgically available peripancreatic, celiac, or superior mesenteric lymph nodal metastases in a separate category (N2) for consideration and study of more extensive, more risky, and more controversial surgical procedures.)

The availability of this surgically realistic classification should help to focus and clarify the debate about the extent of surgery indicated for early gallbladder cancer. Because tumors can spread from lymphatics within the gallbladder wall (increasing incidence with deeper invasion), lymphadenectomy is advisable even for T1 and T2 lesions. Also, because invasion of the liver may not be grossly evident, removal of some contiguous liver tissue is advisable. Thus, the debate about aggressive surgery for *early* gallbladder cancer can be restricted to Stage I, II, and III (T1, T2, or T3, N0 or N1, M0) lesions.

The fact that extending simply cholecystectomy to include spread to adjacent liver and nearby lymph nodes is not new, leads me to review briefly an institutional and personal experience. I have just looked at the original illustrations that Russell Drake made of a gallbladder being removed with a wedge of adjacent liver and a skeletonized hepatoduodenal ligament. They were made just 40 years ago at the request of my mentor, John Waugh. The concept of such extended cholecystectomy and regional lymphadenctomy in relation to modes of spread was refined ten years later by my then senior colleague, Deward Ferris, and others[6].

In my own review of gallbladder cancers treated surgically at the Mayo Clinic between 1960 and 1973[7], I found two patients to add "to the small list of patients reported in the literature to have survived after extirpation of involved tissues outside the gallbladder." I became personally convinced of the value of extended operations, but could conclude then only that "further clinical trial of operations based upon known modes of (early) spread was justified."

Additional institutional experience (1972-84) was reviewed by John Donohue and others[8]. Analysis was hampered by unavailability of surgically relevant staging systems. However, when "patients with transmural tumor infiltration or nodal metastases treated with cholecystectomy ($n$ = 14) or radical surgery ($n$ = 17) were compared, there was a significant difference in survival ($p$ .001)." There were no five-year survivors after cholecystectomy alone, whereas 29 percent of patients were alive five years following radical cholecystecomy. The authors did "feel justified in continuing to pursue this aggressive approach."

Evidence to support the efficacy of extended cholecystectomy and lymphadenectomy is accumulating more rapidly now[9,10,11]. David Nagorney and Geoffrey Thompson have brought Foster's collective literature review of 1987 up-to-date[11]. They found that "the use of extended procedures has risen steadily from 0.5 percent in 1970, 2.1 percent in 1987, to 13.5 percent at the present time." They believe that critical analysis based on the now surgically relevant TNM staging system is likely to provide sound statistical evidence of the efficacy of aggressive surgical treatment in patients with early carcinoma of the gallbladder in the near future.

This commentary is long. You may wonder why, when I agree with so many of the authors' conclusions. I accepted the invitation to comment because I disagree with their reasoning, and because the authors' commentary is too short. I write from retirement, driven by the fear that another generation of surgeons may be taught to think in some wrong way. The authors say that "it is surprising that our patient survived for so long." I am surprised that they are surprised by long survival of a patient whose very early cancer could have been cured by simple cholecystectomy. I am surprised that they attribute the favorable outcome to "aggressive surgical treatment." I am surprised that the authors did not comment upon the risk (or lack of risk) involved in division of the right hepatic artery. I am surprised that they did not mention the option of removing segments IVB and V without removing the common duct as other authors[10,11] have done in this circumstance.

In the final paragraph of the Hammersmith report, the authors correctly state that segmental liver resection, based upon precise knowledge of segmental anatomy and knowledge of modes of spread, can have advantage over wedge resection. I agree, but am concerned about the good surgeon without special experience in hepatic surgery, who is shamed or pushed into doing the good operation poorly. Nathan Womak observed that a good operation done poorly (or unsafely) is not still good. Lymphadenectomy with wedge resection is not really a poor operation, and when liver invasion is not evident grossly, wedge resection of the gallbladder bed, however non-anatomical, involves little risk, and most often will give margins free of tumor. The operation, however ungraceful, is unlikely to become life-threatening, and its safety can be increased by temporary stenting of the ductal bifurcation during operation.

In the hands of a surgeon inexperienced with major hepatic resections, this most often is a better choice than simple cholecystectomy and referral, which occasions more expense, more operative risk, and very little therapeutic advantage.

I am grateful to the authors for giving me an opportunity to reminisce.

Professor Martin Adson
Mayo Clinic
Rochester, Minnesota
USA

*References*

1. Brasfield, R.D. (1961) Right Hepatic Lobectomy for Carcinoma of the Gallbladder: A Five-Year Cure. *Ann. Surg.*, **153**, 563–566 (April)
2. Foote, F. (1961) Personal Communication
3. Beahrs, O.H., Henson, D.E., Hutter, R.V.P. and Kennedy, B.J. (1992) Manual for Staging of Cancer (4th ed). American Joint Committee on Cancer — Philadelphia, JB Lippincott Company, p. 94
4. Adson, M.A. (1986) Clinical Surgeons who Write — Pride, Prejudice and Responsibility. *Arch. Surg.*, **121**, 509 (May)
5. Pack, G.T. and Brasfield, R.D. (1962) Right Hepatic Lobectomy for Cancer of the Gallbladder. In: Pack, G.I., Ariel, I.M. (eds): *Treatment of Cancer and Allied Diseases*, 2nd ed, New York: Paul B. Hoeber

6. Fahim, R.B., McDonald, J.R., Richards, J.C. and Ferris, D.O. (1962) Carcinoma of the Gallbladder: A Study of its Modes of Spread. *Ann. Surg.*, **136**, 114–124
7. Adson, M.A. (1973) Carcinoma of the Gallbladder. *Surg. Clin. N. Amer.*, **53**, 1203 (October)
8. Donohue, J.H., Nagorney, D.M., Grant, C.S., Tsushima, K., Ilstrup, D.M. and Adson, M.A. (1990) Carcinoma of the Gallbladder: Does Radical Resection Improve Outcome? *Arch. Surg.*, **125**, 237 (February)
9. Foster, J. (1987) Carcinoma of the Gallbladder. In: *Surgery of the Gallbladder and Bile Ducts*, Way, L.W., Pellegrini, C.A. (eds). Chapter 31. Philadelphia: WB Saunders Company, pp. 471–485
10. Gall, F.P., Kockerling, F., Scheele, J., Schneider, C. and Hohenberger, W. (1991) Radical Operations for Carcinoma of the Gallbladder: Present Status in Germany. *World J. Surg.*, **15**, 328–336
11. Nagorney, D.M. and Thompson, G. In: *Surgery of the Biliary Tract*, J. Toouli (ed). Edinburgh: Churchill Livingstone (in press)

